# Use of Cone Beam Computed Tomography in Endodontics

**DOI:** 10.1155/2009/634567

**Published:** 2010-03-31

**Authors:** William C. Scarfe, Martin D. Levin, David Gane, Allan G. Farman

**Affiliations:** ^1^Division of Radiology and Imaging Science, Department of Surgical and Hospital Dentistry, The University of Louisville School of Dentistry, 501 South Preston Street, Louisville, KY 40292, USA; ^2^Department of Endodontics, School of Dental Medicine, University of Pennsylvania, Philadelphia, PA 19103, USA; ^3^Kodak Dental Imaging/Practiceworks, 1765 The Exchange, Atlanta, GA 30339, USA

## Abstract

Cone Beam Computed Tomography (CBCT) is a diagnostic imaging modality that provides high-quality, accurate three-dimensional (3D) representations of the osseous elements of the maxillofacial skeleton. CBCT systems are available that provide small field of view images at low dose with sufficient spatial resolution for applications in endodontic diagnosis, treatment guidance, and posttreatment evaluation. This article provides a literature review and pictorial demonstration of CBCT as an imaging adjunct for endodontics.

## 1. Introduction

Since Kells first reported the usefulness of visualizing a lead wire in a root canal on a “radiogram” in establishing the length of a root canal in 1899 [1, 2], radiography has been a pivotal tool in the practice of endodontics. Almost a century later, building on the pioneering efforts of those using conventional computed tomography (CT) and micro-CT, the introduction of maxillofacial CBCT in 1996 provided the first clinically practical technology demonstrating application of 3D imaging for endodontic considerations [3]. 

## 2. Role of Imaging in Endodontics

Radiography is essential to successful diagnosis of odontogenic and nonodontogenic pathoses, treatment of the pulp chamber and canals of the root of a compromised tooth via intracoronal access, biomechanical instrumentation, final canal obturation, and assessment of healing. Imaging serves at all stages in endodontics [4].

1. *Preoperative Assessment*. Imaging achieves visualization of dental and alveolar hard tissue morphology and pathologic alterations to assist correct diagnosis. It provides information on the morphology of the tooth including location and number of canals, pulp chamber size and degree of calcification, root structure, direction and curvature, fractures, iatrogenic defects, and the extent of dental caries. The effects of periradicular and periapical disease can be determined, including the degree of root resorption and characteristics of periapical osteolysis. Larger lesions, only determined by imaging, may necessitate adjunctive surgical procedures in addition to conventional intracanal therapy. Diagnostic radiographs help predict the potential for complications, permit root fracture detection, and demonstrate periapical lesions.
2. *Intraoperative*. During therapy two intraoral periapical images may be performed. The first is a “working” radiograph achieved by placement of a metallic file(s) into the root canal(s) to a length that approximates that of the root as radiological and anatomic root apexes are almost never coincident. This ensures that mechanical debridement of the intracanal contents extends to the apical terminus of the canal and that obturation is dense, homogeneous, and contained within the root canal system. In addition, prior to final obturation, a “final” or pre-condensation radiograph is made to assure proper fitting of the master cone.
3. *Postoperative*. A “postoperative” radiograph immediately after root canal obturation is made to assess the sealing condensation and containment of the root canal filling material within the root canal system. In cases where periradicular healing is incomplete, it acts as a baseline for assessment of healing in the medium and potentially long term. Imaging is important in evaluating the results of previous therapy, delayed healing, evaluating potential obstacles to retreatment, as well as surgical considerations [5].

## 3. Limitations of Conventional 2D Imaging

Intraoral radiography is based on the transmission, attenuation, and recording of X-rays on an analog film or digital receptor, and requires optimized geometric configuration of the X-ray generator, tooth, and sensor to provide an accurate projection of the tooth. The image produced is a two-dimensional (2D) representation of a three-dimensional (3D) object. If any component of the imaging chain process is compromised, the resulting image may demonstrate exposure or geometric errors [6] and be suboptimal. 3D characteristics such as complex dental anatomy and surrounding structures can make interpretation of 2D “shadows” difficult and can contribute to nonhealing of endodontic cases. 

Success in endodontics is assessed in healing of the periapical bone adjacent to obturated canals. Goldman et al. [7] showed that in evaluating healing of periapical lesions using 2D periapical radiographs there was only 47% agreement between six examiners. Goldman et al. [8] also reported that when those same examiners evaluated the same films at two different times, they only had 19%–80% agreement between the two evaluations. 

## 4. Cone Beam Computerized Tomography

In fields of dentistry where 3D imaging is necessary, CBCT is considered by some to be the standard of care [9–14]. CBCT is accomplished by using a rotating gantry to which an X-ray source and detector are fixed. A divergent pyramidal- or cone-shaped source of ionizing radiation is directed through the middle of the area of interest onto an area X-ray detector on the opposite side of the patient. The X-ray source and detector rotate around a fixed fulcrum within the region of interest (ROI). During the exposure sequence hundreds of planar projection images are acquired of the field of view (FOV) in an arc of at least 180°. In this single rotation, CBCT provides precise, essentially immediate and accurate 3D radiographic images. As CBCT exposure incorporates the entire FOV, only one rotational sequence of the gantry is necessary to acquire enough data for image reconstruction. CBCT is a complementary modality for specific applications rather than a replacement for 2D imaging modalities [9–13]. 

The Food and Drug Administration (FDA) approved the first CBCT unit for dental use in the United States in March 8, 2001—the NewTom DVT 9000 (Quantitative Radiology srl, Verona, Italy). FDA approval for three more CBCT units quickly followed in 2003 followed for the 3D Accuitomo, (J. Morita Mfg. Corp., Kyoto, Japan) in March 6, the i-CAT (Imaging Sciences International, Hatfield, PA) in October 2, and the CB MercuRay (Hitachi, Medical Corp., Kashiwa-shi, Chiba-ken, Japan) on October 20. Since 2003, a number of other CBCT units have been FDA approved in the United States, including the Kodak 9000 3D, (Carestream/Trophy, Marne-la-Vallée, France), which is currently the highest resolution unit (Table 1). Several additional units are in various stages of development, testing, or application for FDA approval.

### 4.1. Types of CBCT Equipment

CBCT systems can be categorized according to the orientation of the patient during image acquisition, the scan volume irradiated, or the clinical functionality.

**Patient Positioning**

Depending on the system employed, maxillofacial CBCT can be performed with the patient in three possible positions: (1) sitting, (2) standing, and (3) supine. Equipment that requires the patient to be supine has a larger physical footprint and may not be readily accessible for patients with physical disabilities. Standing units may not be able to be adjusted to a height to accommodate wheelchair bound patients. Seated units are the most comfortable; however fixed seats may not allow ready scanning of physically disabled or wheelchair bound patients. As scan times are often similar to or greater than those used with panoramic imaging, perhaps more important than patient orientation is the head restraint mechanism used.

**Scan Volume**

The dimensions of the FOV, or scan volume, are primarily dependent on the detector size and shape, beam projection geometry, and the ability to collimate the beam. The shape of the FOV can be either a cylinder or spherical (e.g., NewTom 3G). Collimation of the primary X-ray beam limits x-radiation exposure to the region of interest (ROI). Field size limitation therefore ensures that an optimal FOV can be selected for each patient based on disease presentation and the region designated to be imaged. Based on available or selected scan volume height, the use of units can be designed as follows:

1. localized region (also referred to as *focused, small field* or, *limited field*)—approximately 5 cm or less,
2. single arch—5 cm to 7 cm,
3. inter-arch—7 cm to 10 cm,
4. maxillofacial—10 cm to 15 cm,
5. craniofacial—greater than 15 cm.

In general, the smaller the scan volume, the higher the spatial resolution of the image. As the earliest sign of periapical pathology is discontinuity in the lamina dura and widening of the periodontal ligament space, it is desirable that the optimal resolution of the any CBCT imaging system used in endodontics does not exceed 200 *μ*m—the average width of the periodontal ligament space. The 3D Accuitomo (J. Morita, Corporation, Kyoto, Japan)—the first of the small FOV systems—provided a resolution of 0.125 mm. At the time of publication, nominal voxel resolution varies from 0.4 mm to 0.076 mm.

**Multimodality**

**Figure 1:**
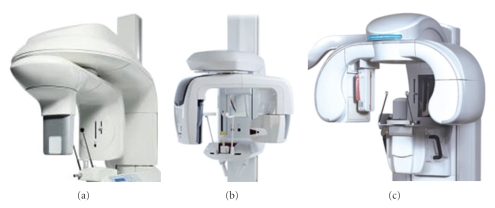
Examples of hybrid CBCT units. (a) KODAK Dental Imaging 9000 3D, (b) Veraviewepocs 3D, and (c) Picasso Trio.

Hybrid multimodal systems combine digital panoramic radiography with a relatively small-to medium-FOV CBCT system. This combination is now priced at a level similar to upper-level digital panoramic radiographic systems of the relatively recent past. Cost savings come from the fact that the cost of CBCT detectors is highly dependent on size. The ProMax 3D CBVT (Planmeca Oy, Helsinki, Finland) was the first to incorporate a small FOV 3D sensor to their ProMax digital panoramic line, which can be also be retrofitted to any of the prior ProMax digital models. Examples of other hybrid units are the Veraviewepocs 3D (J. Morita, Corporation, Kyoto, Japan), the Picasso Trio (Vatech/E. Woo Corporation, Korea), and the Kodak Dental Imaging 9000 DS (Kodak Dental Imaging/Practiceworks Atlanta, GA, USA) (Figure 1).

There are advantages beyond reduced capital costs to small FOV CBCT units for endodontic applications. First, a small FOV means that high-resolution images with a spatial resolution down to 0.076 mm isotropic voxel size can be achieved at very low exposure to ionizing radiation and without extensive reconstruction times that would be expected with larger FOV systems due to the greater file sizes to be processed. Second, a restricted FOV reduces the volume examined, and for which the practitioner is responsible to interpret. Small FOV systems concentrate on the dental arches or individual temporomandibular joints, the structures in which the average dentist is most familiar. There is less detail of the cranial cavity, paranasal sinuses, ear, and neck—structures less familiar to the average dentist. A small FOV CBCT system is undoubtedly too restrictive for maxillofacial surgeons who conduct craniofacial and orthognathic surgery or for complex implant/prosthetic situations where the jaws and both temporomandibular joints are best evaluated in toto rather than as individual components; however, third-party software is now available to “stitch” together adjacent small FOV images [15].

### 4.2. Radiation Dose Considerations

For a meaningful comparison of radiation risk, radiation exposures are converted to effective dose (E), measured in Sieverts (Sv). The Sv is a large unit; so in maxillofacial imaging milli-[10^−3^; mSv] or micro-[10^−6^; *μ*Sv] Sieverts are reported. The radiation dose to specific tissues is measured, adjusted for the amount of that tissue in the field of view, and weighted based on radiation sensitivity of the tissue. The weighted tissue/organ doses are then summed to assess Effective Dose (E). Comparisons can be performed with respect to natural background radiation. 

The tissues/organs used to calculate the effective dose are specified by the International Commission on Radiological Protection (ICRP). The organs used to calculate effective dose for imaging of the head include the bone marrow, thyroid, esophagus, skin, bone surface, salivary glands, brain, and “remainder” tissues [16]. Published effective doses for digital panoramic radiographs range from 5.5 to 22.0 *μ*Sv [17], while digital cephalometric radiographs have effective doses of 2.2 to 3.4 *μ*Sv [18]. This compares with an average annual effective dose from background radiation in the United States of about 3,000 *μ*Sv (3.0 mSv).

There are a number of factors that will affect the radiation dose produced by a CBCT system: the imaging parameters used (kVp, mAs); pulsed beam versus continuous beam; amount, type, and shape of the beam filtration; the number of basis images dependent partly on use of 360° or lesser rotations; and limitations on the size of the field of view. Factors such as beam quality and filtration are unique to a specific machine, while other factors, such as FOV, can sometimes be operator controlled. Typically, the smaller the field of view for a given system, the lower the radiation dose applied [19, 20]. Since the effective dose is computed from a weighted summation of doses to various organs, removing some organs from the path of the X-ray beam can reduce the effective dose. Since the radiation received by the thyroid gland contributes a large amount to the effective dose, limiting the beam to the maxilla instead of the whole head produces a lower effective dose. 

Tables 2 and 3 provide the most recent published radiation exposures for selected CBCT units using ICRP (2007) recommendations [19–30] and compares them as multiples of digital panoramic examinations (using an average digital panoramic exposure of 14 *μ*Sv obtained from the published range of effective dose) and equivalent days of per capita background dose (based on an annual full body background exposure of 3 mSv). At the time of publication, the CBCT unit with the highest resolution and the smallest field of view (the KODAK 9000 3D) involves patient radiation exposure varying from as little as 0.4 to 2.7 digital panoramic equivalents depending on the part of the mouth studied [30].

### 4.3. Advantages of CBCT in Endodontics

Perhaps the most important advantage of CBCT in endodontics is that it demonstrates anatomic features in 3D that intraoral, panoramic, and cephalometric images cannot. CBCT units reconstruct the projection data to provide interrelational images in three orthogonal planes (axial, sagittal, and coronal). In addition because reconstruction of CBCT data is performed natively using a personal computer, data can be reoriented in their true spatial relationships. 

**Figure 2:**
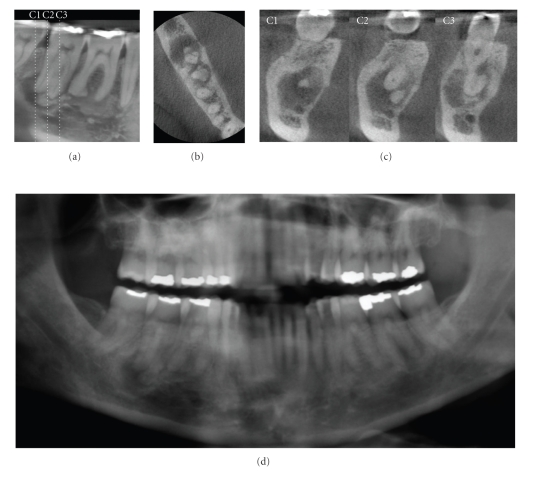
A 52-year-old Caucasian female was referred for assessment of multiple periapical areas associated with the mandibular right first and second molars. Curved planar (a), axial (b), and correlated multiple cross-sectional (c) images are shown. There are areas of mixed central opacity and peripheral radiolucency associated with the apices of the teeth; however no expansion, tooth resorption, or displacement is evident. Hypercementosis is observed on the distal root of the first molar. On clinical examination, all teeth in this quadrant tested vital. Based on a working diagnosis of florid cemento-osseous dysplasia, an additional digital panoramic radiograph ((d) cropped panoramic image) was performed and revealed similar bony patterns in the left posterior maxilla and mandible. Management of this patient comprised a 6-month recall comparison of focused CBCT images to judge the progression of the lesion.

Due to the isotropic nature of the constructed volume elements (“voxels”) constituting the volumetric dataset, image data can be sectioned nonorthogonally. Most software provides for various nonaxial 2D images in multiplanar reformation (MPR). Such MPR modes include oblique, curved planar reformation (providing “simulated” distortion free panoramic images) and serial transplanar reformation (providing cross-sections), which can be used to highlight specific anatomic regions for diverse diagnostic tasks (Figure 2). Enhancements including zoom magnification, window/level adjustments, and text or arrow annotation can be applied. Cursor-driven measurement algorithms provide the clinician with an interactive capability for real-time dimensional assessment. On-screen measurements are free from distortion and magnification.

Because acquisition occurs innately as high-resolution three-dimensional volumetric data and can be displayed as interactive images, CBCT technology provides the clinician with an unparalleled visualization of the often complex relationships and boundaries between teeth and their associated pathology and anatomic features within the alveolus and jaws such as the maxillary sinus and mandibular canal and foramen.

### 4.4. Limitations of CBCT in Endodontics

 Despite the provision of the third dimension, the spatial resolution of CBCT images (0.4 mm to 0.076 mm or equivalent to 1.25 to 6.5 line pairs per mm^−1^[lp.mm^−1^]) is inferior to conventional film-based (approx. 20 lp.mm^−1^) or digital (ranging from 8–20 lp.mm^−1^) intraoral radiography [31]. However, the ability of this technology to demonstrate geometrically accurate images in all three dimensions and the elimination of anatomic noise facilitates the assessment of a number of features important in endodontic diagnosis, treatment, and long-term management. The optimal resolution for CBCT images in endodontics is invariably task specific—however; most aspects of endodontics involve imaging of small structures. Liedke et al. [32] have recommended a minimal voxel resolution of 0.3 mm for the detection of external root resorption. Ex vivo research performed at our institution [33] has determined the effect of isotropic voxel dimensions on observer detection of the presence or absence of secondary canals in the mesiobuccal root of the maxillary first permanent molar. Observer interrater reliability and detection of mesiobuccal canals increased substantially with increasing resolution with more than 93% accuracy with a voxel resolution of 0.12 mm but accuracy barely over 60% with 0.4 mm resolution. The diagnosis of other subtle conditions (e.g., initial stages of apical periodontitis) involving the periodontal ligament space, which has an average dimension of 0.2 mm, also demands high resolution. 

The CBCT projection geometry results in the whole volume within the FOV being irradiated with every basis image projection. Scattered radiation is produced omnidirectionally and is recorded by pixels on the cone beam CT detector but does not reflect actual attenuation of the object within a specific path of the X-ray beam. Additional recorded X-ray nonlinear attenuation is *noise*. This can be eliminated somewhat by algorithms such as wavelet transformation of filtered back-projection data; however, because of the use of an area detector, some of this nonlinear attenuation is recorded and contributes to image degradation when not adequately attended to by noise reduction algorithms. Remaining noise contributes to the graininess of the image which can be more pronounced in images in systems using a large FOV, especially where low signal due to restricted radiation exposure is the case.

Maxillofacial CBCT images presently lack the ability to record subtle changes in attenuation across a wide range of tissue radiodensities. In endodontics, contrast resolution might well be of importance in distinguishing the nature of periapical or sinus soft tissue contents. Three factors, inherent in the CBCT acquisition process, presently limit contrast resolution: (1) scattered radiation contributing to the potential for increased noise, (2) CBCT systems pronounced “heel effect” due to the divergence of the X-ray beam over the area detector producing nonuniformity of the incident X-ray beam, and (3) detector imperfections affecting linearity in response to x-radiation. These factors, and a desire to restrict dose, contribute to restricting the application of current maxillofacial CBCT imaging to the assessment of osseous structures. Work continues to develop systems capable of a wide contrast range supporting both hard tissue and soft tissue applications while still limiting dose.

CBCT images, like those from other diagnostic modalities, are susceptible to artifacts that affect image fidelity. Artifacts can be attributed to four sources [34]: (1) the patient; (2) the scanner; (3) artifacts specific to the CBCT system used including partial volume averaging, undersampling, and the cone beam effect; and (4) X-ray beam artifacts arising from the inherent polychromatic nature of the projection X-ray beam that results in what is known as *beam hardening* (i.e., mean energy increases because lower energy photons are absorbed in preference to higher-energy photons). Beam hardening results in two types of artifact: (1) distortion of metallic structures due to differential absorption, known as a *cupping artifact*; and (2) streaks and dark bands that can appear between two dense objects. The presence of dental restorations, including apically positioned retrograde restorations, in the FOV can lead to severe streaking artifacts. As the CBCT X-ray beam is heterochromatic and has lower mean kVp energy compared to conventional CT, such artifact can be pronounced in CBCT images. In clinical endodontic practice, CBCT scanners with a limited field of view might provide clearer images as they can avoid scanning structures outside the region of interest susceptible to beam hardening (e.g., metallic restorations, dental implants).

## 5. CBCT Applications in Endodontics

A PUBMED search performed in May 2009 (search terms: cone beam, CBCT, endodontics, root canal, periapical) resulted in less than 30 comparative retrospective or ex vivo studies published quantifying specific clinical efficacies of CBCT imaging in endodontics. Similarly a recent review performed by the SEDENTEXCT project indicated that while several nonsystematic reviews in the literature provide a favorable perspective of the role of CBCT imaging in endodontics, only a few studies have been published that satisfy the criteria for formal systematic review [35]. 

**Figure 3:**
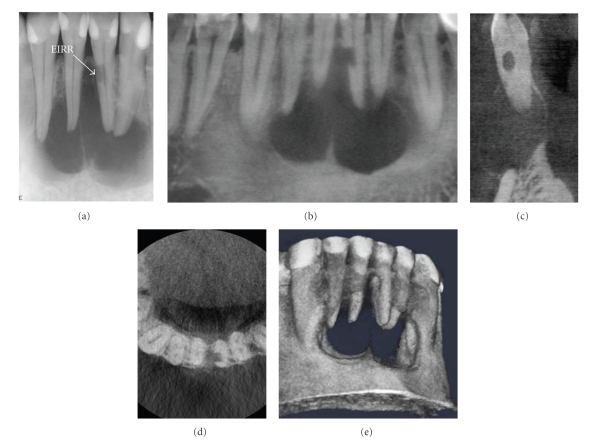
After suffering a traumatic blow from a soccer ball six years earlier, a 28-year-old male presented with a soft, convex-shaped indurated buccal and lingual swelling in the mandibular symphyseal region. A periapical image (a) showed a large, multilocular lesion. External inflammatory root resorption (EIRR) was noted on the mandibular left lateral incisor. All four mandibular anterior teeth tested nonvital. CBCT images ((b) curved planar, (c) cross-sectional, (d) axial, (e) 3D reconstruction) showed that the resorptive lesion was extended from the root canal space to the periodontal membrane, necessitating repair or extraction; no exploratory procedure was necessary to determine the extent of the defect.

**Figure 4:**
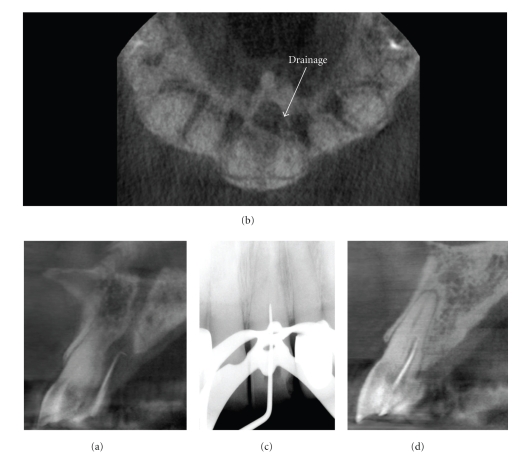
A 79-year-old male was referred for endodontic treatment of the maxillary left central incisor after a palatal sinus tract was noted. CBCT imaging was initially performed with a gutta percha cone marker inserted into the sinus tract to determine the source of the infection. Sagittal images (a) demonstrated that the lesion terminated at the periapex of the maxillary left central incisor after coursing through the incisive canal whereas drainage was visible on the axial image (b). Perioperatively, only the mesiodistal direction could be determined on conventional intraoral periapical radiography (c) and treatment suspended when the explorer reached 17 mm because of the danger of perforation in the facial or palatal direction. Subsequent cross-sectional perioperative CBCT imaging (d) with an intracanal gutta percha marker indicated that the initial access preparation was directed palatally. Correction of the access facially resulted in gaining access to the apical terminus; treatment was completed without complication.

While there are presently no definitive patient selection criteria for the use of CBCT in endodontics, the use of CBCT in endodontic diagnosis should not be avoided or ignored. One of the authors (Martin D. Levin) is a Board Certified Endodontist with a full time private practice with limited field CBCT. CBCT has been used to assist diagnosis and facilitate treatment in more than half of all patients referred to his practice for assessment and treatment of complex endodontic conditions (Figures 3 and 4). 

**Figure 5:**
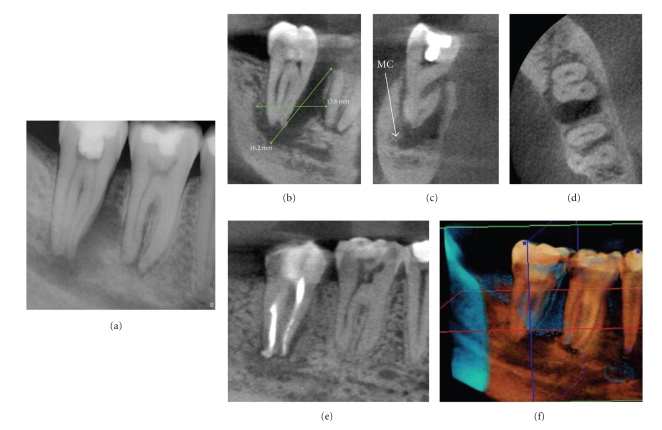
Treatment of large destructive lesions associated with pulpal pathosis benefit from 3D imaging providing better visualization of the spatial relationships of the tooth to anatomic landmarks and canal morphology. This patient presented with mild dysthesia of the right mandibular dentition. Conventional periapical imaging (a) demonstrated a large apical and mesial ill-defined rarefaction associated with the right mandibular second molar in close proximity to the inferior alveolar nerve. CBCT images ((b) sagittal, (c) cross-sectional, (d) axial) demonstrate the proximity of the lesion to the mandibular canal (MC). Therefore during treatment great care was taken to prevent obturation material extrusion past the apical terminus and possible traumatization of the IAN. Additional parasagittal CBCT images document the progression of healing at 6 months (e). In addition digital subtraction composite 3D imaging at 6 months (f) provides three-dimensional visualization of healing. (Data acquired at 0.076 mm resolution on an KODAK Dental Imaging 9000 DS (Dental Imaging/Practiceworks Atlanta, GA, USA) and 3D subtraction composite reformatted using InVivo Dental (Anatomage, San Jose, CA)).

**Figure 6:**
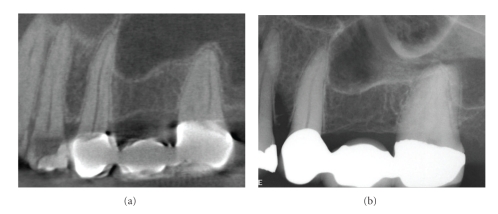
A female patient presented with a twenty-year history of mostly continuous, unilateral, poorly localized severe aching of the maxillary left quadrant. The pain was not associated with sensory loss or other physical signs and pulp tests, and conventional imaging studies were within normal limits. Clinically there was no cessation of pain after administration of local anesthetic. This neuropathic pain syndrome, initially termed atypical facial pain, is more recently known as persistent idiopathic facial pain (PIFP). PIFP refers to pain along the territory of the trigeminal nerve that does not fit the classic presentation of other cranial neuralgias. Diagnostically challenging, PIFP is frequently misdiagnosed and is often attributed by patients to dental procedures, facial trauma, and rarely, by some clinicians, as Neuralgia-Inducing Cavitational Osteonecrosis (NICO). Dynamic visualization of sequential curved planar parasagittal CBCT-reformatted images at 0.076 mm thickness (a) confirmed the absence of obvious pathosis of odontogenic origin as diagnosed from the original intraoral periapical of the region (b). Note the radiolucent area within the coronal portion of the first molar under the radiopaque disto-occlusal restorative material; this represents a streak artifact due to “photostarvation” in the horizontal plane due to the attenuation of adjacent amalgam and radiopaque material and subsequent reduction in available data for image reconstruction. A negative CBCT imaging finding is often very reassuring for these unfortunate patients who often question a nonodontogenic diagnosis. Psychiatric symptoms of depression and anxiety are prevalent in this population and compound the diagnostic conundrum.

Depending on the equipment used, CBCT exposure may subject a patient to only slightly higher radiation doses than conventional 2D imaging—or considerably more, so it is important that practitioners follow professional judgment in minimizing the radiation dose to the patient to that deemed essential for optimal diagnosis and treatment guidance. There should be justification of the exposure to the patient such that the total potential diagnostic benefits are greater than the uncertain detriment radiation exposure might cause. Published research, while admittedly sparse, indicates that CBCT has several applications in selected endodontic cases (Figures 5 and 6). The absence of high prospective randomized clinical trials underlines the need for further research on the treatment outcomes related to CBCT applications in endodontic practice. At this time CBCT should not be considered a replacement for standard digital radiographic applications. Rather, CBCT is a complementary modality for specific applications [35].

### 5.1. Preoperative Assessment

#### 5.1.1. Tooth Morphology

**Figure 7:**
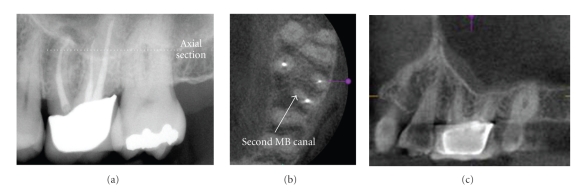
Maxillary first and second molars often present treatment challenges because of the frequent presence of mesioaccessory (mb2) canals. On initial periapical radiographic examination of this previously treated maxillary left first molar, no mb2 canal was detected; however a periapical lesion is seen (a). Note the overlap of the interproximal contacts between the molars indicating a geometric distortion in the horizontal plane. CBCT imaging ((b) 0.076 mm axial and (c) 0.076 parasagittal) clearly demonstrates an additional canal that was not previously treated.

The success of endodontic treatment depends on the identification of all root canals so that they can be accessed, cleaned, shaped, and obturated [36]. The prevalence of a second mesiobuccal canal (MB2) in maxillary first molars has been reported to vary from 69% to 93% depending on the study method employed. This variability occurs in the buccolingual plane where superimposition of anatomic structures impedes detection of small structural density changes [37, 38]. Conventional radiographic techniques, at best, can only detect up to 55% of these configurations (Figure 7) [39]. Ramamurthy et al. [40] found that raters evaluating different two-dimensional film modalities were rarely able to detect more than a 50% presence of MB2 canals. They found differences in detection rates with complementary metal oxide semiconductors (CMOSs), analog film, and photostimulable phosphor plates (PSP) detecting 55%, 44%, and 39% of MB2 canals, respectively. Matherne et al. [41] compared the ability of three board-certified endodontists to detect the number of root canals on intraoral digital (both charged-couple device and photostimulable phosphor) plate images with CBCT in 72 extracted teeth (3 equal groups of maxillary molars, mandibular premolars, and mandibular incisors). They found that on average the observers failed to detect at least one root canal in 40% of teeth using intraoral radiographs. CBCT evaluations identified an average of 3.58 root canals (RCS) per maxillary molar, 1.21 per mandibular premolar, and 1.5 per mandibular incisor. Evaluation of CCD images demonstrated an average number of 1.0 RCS per mandibular incisor, 1.0 per mandibular first premolar, and 3.1 per maxillary molar. Evaluation of PSP images demonstrated an average number of 1.3 RCS per mandibular incisor, 1.1 per mandibular first premolar, and 3.0 per maxillary molar. Baratto Filho et al. [42] investigated the internal morphology of extracted maxillary first molars by comparing detection rates obtained using an operating microscope and CBCT to ex vivo sections. They reported an ex vivo prevalence of a fourth canal in 67.14% of teeth and additional root canals in 92.85% of mesiobuccal roots. Clinical assessment provided slightly lower overall (53.26%) but higher (95.63%) MB2 detection rates whereas CBCT results showed the lowest overall (37.05%) detection rate. They indicated that CBCT provided a good method for the initial evaluation of maxillary first molar internal morphology but that the use of operating microscopes was optimal. Unpublished ex vivo research performed at our institution [33] investigated the effect of increasing voxel resolution on the detection rate of multiple observers of the MB2 on 24 maxillary first molars by CBCT. Compared to the overall prevalence of MB2 (92% prevalence), CBCT detection rates increased from 60% to 93.3% with increasing resolution suggesting that if CBCT is to be used, then resolutions in the order of 0.12 mm or less are optimal.

CBCT imaging has also been reported to characterize the high prevalence of the distolingual canal in Taiwanese individuals [43], highlight anomalies in the root canal system of mandibular premolars [44], and assist in the determination of root curvature [45].

#### 5.1.2. Dental Periapical Pathosis

**Figure 8:**
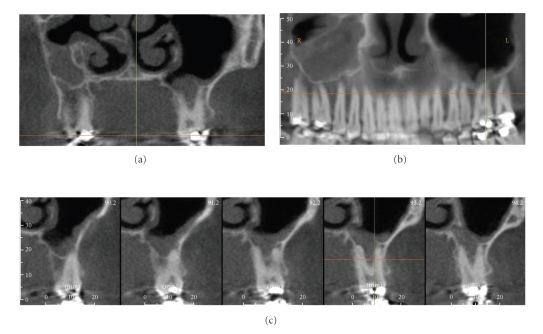
On conventional intraoral periapical radiography periapical mucositis (PM) presents as a relatively radiopaque, soft-tissue, dome-shaped lesion localized to the apex of a maxillary posterior tooth and projecting into the floor of the maxillary sinus. Most are indistinguishable from mucosal lesions of intrinsic sinus origin such as antral mucosal pseudocysts (see Figure 9). While clinically asymptomatic, they are usually associated with necrotic or failing root canal-filled teeth. PM is a localized mucosal thickening of the sinus membrane, secondary to a breach of periradicular inflammation, and will resolve after successful endodontic treatment. This patient gave a history of persistent left side pain over the maxillary molar region of 4-month duration. Treatment for sinusitis did not relieve the symptoms. Panoramic and intraoral dental radiology did not reveal a cause. Coronal CBCT imaging (a) demonstrates acute sinusitis bilaterally with 50% to 70% opacification and previous uncinectomy and antrostomy (as evidenced by the loss of the superior medial wall of the right sinus) of the right sinus, whereas the left sinus shows thickened mucosal lining with a dome-shaped soft tissue lesion overlying the roots of the restored left maxillary first molar tooth. 5 mm reformatted panoramic (b) and 1 mm cross-sectional (c) reconstructions of the maxillary left first molar show periapical lesional penetration and communication with the floor of the sinus in this region. Data acquired on an iCAT, Imaging Sciences International, Hatfield, PA USA at 0.3 mm resolution and reformatted using InVivo Dental, Anatomage, San Jose, CA.

**Figure 9:**
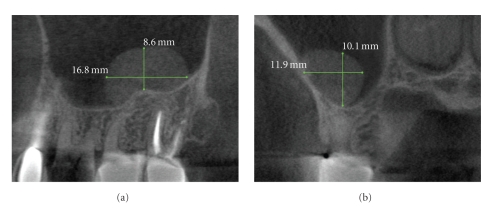
Antral mucosal pseudocysts, also called mucus retention cysts, are a relatively common localized dome-shaped antral mucosal swelling, often of allergic origin and while they can occur anywhere in the sinus present diagnostic challenges when associated with the floor of the maxillary sinus. CBCT imaging ((a) sagittal, (b) cross-sectional) can be useful in differentiating these lesions from periapical mucositis in that the former is usually not associated with disruption of the floor of the sinus and expansion superiorly from the apex of roots of adjacent teeth.

**Figure 10:**
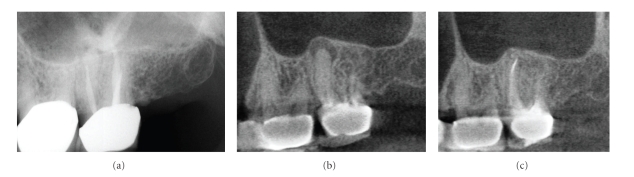
A periapical radiograph of the maxillary left first molar (a) shows an area of low density surrounding the mesial root with incomplete endodontic treatment. A contemporaneous CBCT parasagittal image (b) shows a chronic periradicular osteoperiostitis, or “halo lesion,” where the apical periodontitis has caused displacement of the periosteum but did not penetrate the antral floor. Three months after retreatment, CBCT imaging (c) demonstrates complete apical resolution.

**Figure 11:**
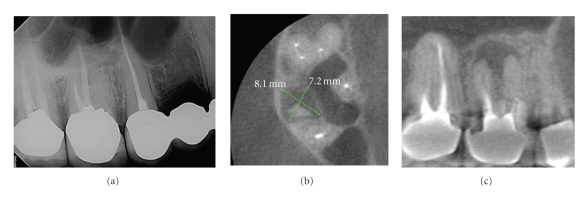
This patient was referred for discomfort and swelling in the maxillary right quadrant. While an initial periapical radiograph (a) clearly demonstrated an untreated mesial root in the maxillary first molar, periradicular periodontitis was undetectable. CBCT images ((a) axial, (b) sagittal) clearly identify a large apical radiolucent lesion associated with the mesial root extending to the distobuccal root. The tooth was retreated and the symptoms subsided.

The most common pathologic conditions that involve teeth are the inflammatory lesions of the pulp and periapical areas (Figures 8, 9, 10, and 11). Lofthag-Hansen et al. [46] compared the accuracy of 3 observers using high-resolution limited FOV CBCT to intraoral radiographic paralleling technique using two images, one with a horizontal tube shift difference of about 10° for the diagnosis of periapical pathology on 46 teeth. While CBCT and intraoral radiographs identified 53 roots with lesions, CBCT identified an additional 33 roots with lesions (62%). Observers agreed that additional clinically relevant material was provided by CBCT imaging in 32 of the 46 (69.5%) teeth imaged. Stavropoulos and Wenzel [47] compared CBCT (NewTom 3G) to digital- and film-based intraoral periapical radiography for the detection of periapical bone defects on 10 frozen pig mandibles by four calibrated examiners. They reported that CBCT provides greater diagnostic accuracy (61%) compared with digital (39%) and (44%) conventional radiographs. Özen et al. [48] performed a similar study comparing the detection of chemically induced periapical lesions by three observers using digital- and film-based conventional radiography to two CBCT systems (Iluma, Imtec Imaging, Ardmore, OK and iCAT, Imaging Sciences International, Hatfield, PA). They found that CBCT systems provided similar intra- and interobserver agreement substantially higher than either conventional radiography. They indicated that while detection rates for CBCT were higher, they did not advocate the replacement of intraoral radiography for detecting periapical lesions in routine clinical practice due to financial and dose considerations.

**Figure 12:**
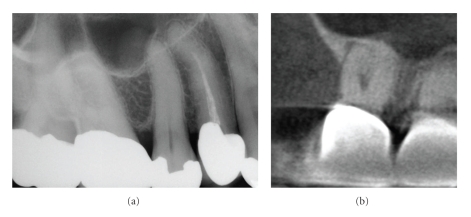
Difficulty in visualizing furcal and apical lesions with periapical radiography due to lack of coverage, anatomic superimposition, and geometric distortion is well established. The periapical radiograph (a) of the maxillary right second molar in an asymptomatic patient is unremarkable; however there is lack of coverage of the second molar posteriorly and marked superimposition of the distal root of the first molar over the mesial root of the second molar. The corresponding cropped sagittal CBCT image (b) of the second molar demonstrates a furcal radiolucency and associated periradicular periodontitis. Subsequent clinical investigation found this tooth to be nonvital.

Estrela et al. [49] compared the accuracy of CBCT, panoramic and periapical radiographs from a consecutive sample of 888 imaging exams of patients with endodontic infection (1,508 teeth) in the detection of apical periodontitis (AP). While a gold standard was not available, they found the detected prevalence of AP to be significantly higher with CBCT (Figure 12). Estrela and colleagues proposed a periapical index based on cone beam-computed tomography (CBCTPAI) for identification of AP [50]. The CBCT PAI is a 6-point (0–5) scoring system calculated from determining the largest lesional measurement in either the buccopalatal, mesio-distal, or diagonal dimension and taking into account expansion and destruction of cortical bone. Using their criteria, 3 observers applied it to 1,014 images (periapical radiographs and high resolution CBCT images) originally taken from 596 patients. They found that CBCT imaging detected 54.2% more AP lesions than intraoral radiography alone. Similar results are reported by Low et al. [51] who compared the preoperative consensus assessment of the apical condition of 37 premolars and 37 molars in the maxilla (156 total roots) using periapical radiography and CBCT referred for possible apical surgery and found the later method to demonstrate significantly more lesions (34%) than conventional radiography. CBCT showed significantly more findings including expansion of lesions into the maxillary sinus, sinus membrane thickening, and missed canals. Using an ex vivo model consisting of 2 mm diameter defects placed in the cancellous bone at the apices of 10 first molar teeth on six partially dentate intact human dry mandibles, Patel et al. [52] reported a detection rate of 24.8% and 100% for intraoral radiography and CBCT imaging respectively. 

The generally higher detection rates afforded by CBCT are similar to those reported for conventional CT [53]. This may be of clinical importance in patients who present with pain or who have poorly localized symptoms associated with an untreated or previously root treated tooth with no evidence of pathology identified by conventional imaging [54–56].

#### 5.1.3. Root Fracture

**Figure 13:**
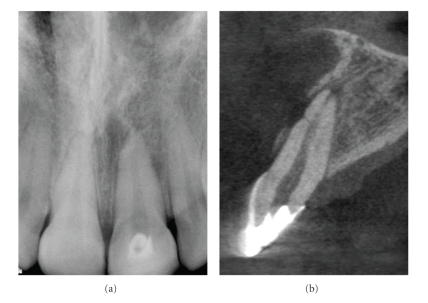
Traumatic injuries to the anterior dentition may result in a horizontal root fracture. Visualizing these fractures with periapical radiographs can be difficult as the beam must be in parallel alignment to the axis of the fracture. Conventional periapical image (a) shows horizontal radiolucent line separating the apical 1/3rd of the root. Note the loss of lamina dura and lateral radiolucency on the distal root surface adjacent to the horizontal radiolucency. Cross-sectional high-resolution (0.076 mm) image showing “V” shaped fracture and minimal displacement of the root segments. Note the loss of buccal cortical plate, widening of the buccal periodontal ligament space and periapical rarefaction.

While root fractures are less common than fractures of the crown and occur in only 7% or fewer of dental injuries [57, 58], they are difficult to diagnose accurately using conventional radiography. Numerous authors have illustrated the usefulness and importance of CBCT in the diagnosis and management in specific aspects of dento-alveolar trauma, especially root fractures (Figure 13) [59–62], luxation and/or displacement, and alveolar fracture [60]. CBCT has found particular application for the diagnosis of root fractures. Hassan et al. [63] compared the accuracy of 4 observers in detecting ex vivo vertical root fractures (VRFs) on CBCT and periapical images and assessed the influence of root canal filling on fracture visibility. They found an overall higher accuracy for CBCT (0.86) scans than periapical radiographs (0.66) for detecting VRF which was slightly reduced by the presence of opaque obturation material. Similar results were reported by Kamburoğlu et al. [64] who compared the diagnostic accuracy of 3 oral and maxillofacial radiologists in detecting simulated horizontal root fractures on conventional radiographic (analog film, PSP and CCD-based digital) images and CBCT of 36 teeth. They found that the sensitivity of CBCT (0.92) was significantly greater than analog film (0.74), PSP (0.71), and CCD (0.68) images. Most recently Bernardes et al. [65] retrospectively compared conventional periapical radiographs and CBCT images for 20 patients with suspected root fractures. They found that CBCT was able to detect fractures in 18 (90%) of patients whereas conventional periapicals could only detect fractures 6 to 8 of the cases (30% to 40%) and indicated that CBCT was an excellent supplement to conventional radiography in the diagnosis of root fractures.

#### 5.1.4. Root Resorption

**Figure 14:**
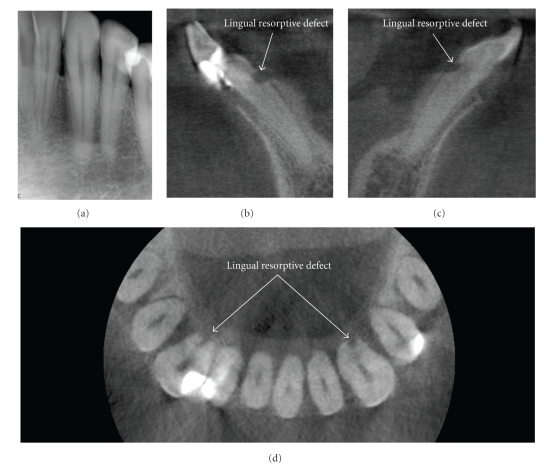
This patient was referred for endodontic revision of the mandibular right lateral incisor. Initial periapical radiographic examination of the mandibular anterior teeth including the left anterior (a) was unremarkable. Note the appearance of a large radiolucency at the on the distal surface of the left canine due to nonperpendicular X-ray beam projection of the distal curvature of the cervical margin. CBCT images ((b) right cross-sectional, (c) left cross-sectional, (d) axial) demonstrated an occult finding of early ERR on the mandibular left and right mandibular cuspids. Early detection and classification of the lesion improve the prognosis and assist in early direct treatment consisting of surgical exposure and removal of granulation tissue from the resorbing lacunae and sealing.

**Figure 15:**
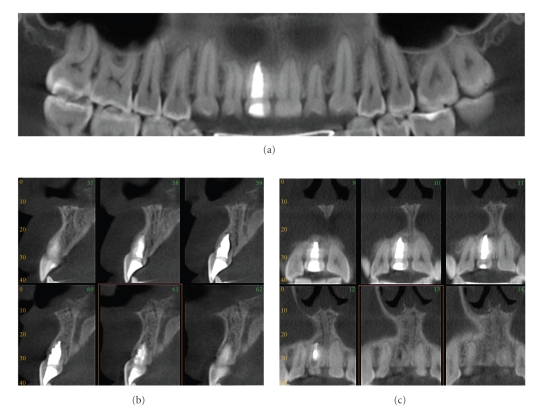
Replacement external resorption associated with root canal filled right maxillary central incisor. An oblique multiplanar reformatted “panoramic” image (a) shows the dentition with minimal restorations and a single root canal filled maxillary right central incisor; note that the obturation is large in relation to the width of the adjacent left maxillary central suggesting endodontic treatment at an early age. Sequential 1 mm cross-sectional (b) and parasagittal (c) images show bone trabecular-like replacement of the superior and palatal aspects of the root indicative of replacement resorption. Initial management consisted of conventional endodontic treatment. Because of the questionable long-term prognosis of the apical resorptive lesions, periodic CBCT imaging is recommended with a view towards surgical revision therapy consisting of apicoectomy and retrograde root canal treatment (data acquired on an iCAT, Imaging Sciences International, Hatfield, PA USA at 0.4 mm resolution and reformatted using InVivo Dental, Anatomage, San Jose, CA).

The use of serial cross-sectional CT in diagnosing the size and location of external root resorption (ERR) has been well described (Figures 14 and 15) [66–68]. Similarly, several authors have presented selected cases illustrating the utility of CBCT in the detection of small lesions, localizing and differentiation the resorption from other conditions, classification of the lesion, in determining prognosis, and directing treatment (Figures 14 and 15) [54, 69–73]. The accuracy of CBCT in the detection of surface defects, while higher than conventional imaging modalities, is not perfect [73] and appears to increase with increasing voxel resolution of the volumetric dataset [30]. CBCT has also been shown to have particular application in the assessment of the postorthodontic apical root resorption [74] and, in particular, of the roots of lateral maxillary incisors by impacted maxillary canines [75–77].

**Figure 16:**
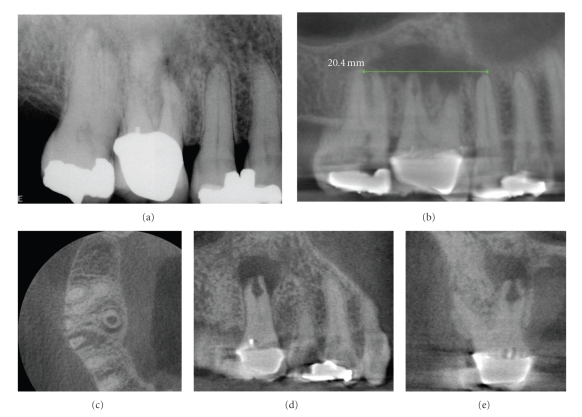
Because two-dimensional imaging suffers from superimposition of anatomic structures, determination of the extent and pathogenesis of periradicular lesions can present diagnostic challenges. This is particularly true of the maxillary posterior region, where the roots of teeth overlap and anatomic structures form complex patterns. A patient presented with discomfort in the maxillary right that extended from the nose to the ear. On clinical examination buccal swelling and induration were present—all teeth tested vital except the maxillary right first molar. A periapical radiograph (a) demonstrated areas of low density at the apices of the maxillary right first and second molars. CBCT images ((b) 10 mm curved planar, (c) axial, (d) sagittal, (e) cross-sectional) however demonstrated a much more extensive (21.4 mm maximum length) unilocular lesion, centered on the palatal root of the maxillary first molar, and extending anteriorly to the second bicuspid and posteriorly to the second molar. Also note the large internal resorptive lesion at the mid-palatal root of the maxillary first molar, not visible on the periapical radiograph. Biopsy confirmed the lesion to be a periapical granuloma with abscess formation.

Internal root resorption (IRR) within the root canal itself is rare, usually asymptomatic, slowly progressing, and presents as a serendipitous finding on intraoral radiographic examination. The inflammatory etiology of the resorptive process is not fully understood, although IRR has been associated with a history of trauma, persistent chronic pulpitis, and as well as orthodontic treatment. It is very common that internal and external inflammatory root resorption are confused and misdiagnosed. Still, accurate assessment is essential as these conditions represent totally different pathological processes, with different etiological factors and treatment protocols. Diagnosis using conventional radiography is difficult; however, unlike external resorption, which presents with irregular radiolucency and intact root canal, internal resorption has clearly defined borders with no canal radiographically visible in the defect (Figure 16) [78]. CBCT has been used successfully to confirm the presence of IRR and differentiate it from ERR [71].

### 5.2. Postoperative Assessment

Monitoring the healing of apical lesions is an important aspect of postoperative assessment in endodontics. Pinsky et al. [79] investigated the accuracy of CBCT (iCAT with 0.2 mm voxel resolution) in the detection of the simulated osseous defects of varying diameters and depths in an acrylic block and on the buccal cortex of a human mandible. They found mean accuracy for the acrylic block to be within the tolerance of the nominal resolution of the CBCT unit (−0.01 mm ±0.02 (SE) mean width difference and −0.03 mm ±0.01 (SE) mean height difference). For the human mandible, they found differences to be slightly higher (mean width accuracy, −0.07 mm (±0.02 SE); mean height accuracy, −0.27 mm (±0.02 SE)). In addition they segmented the defect and applied and automated algorithm to calculate volume. They found that automated volume accuracy error was significantly higher (−6.9 mm^3^ (±4 SE)) than manually derived measurements (−2.3 mm^3^ (±2.6 SE)). 

As adequacy of root canal obturation is an important determinant of endodontic success, it might be considered that CBCT is used in the initial and subsequent monitoring of the integrity of root canal fillings. Soğur et al. [80] compared the subjective quality of 3 radiologists and 3 endodontists using limited field CBCT, storage phosphor plate (SPP), and F-speed analog film images for the evaluation of length and homogeneity of root fillings on 17 extracted permanent mandibular incisor teeth. They found that SPP and F-speed film images were perceived as superior to the corresponding CBCT images and they reported that this may be due to the presence of streaking artifacts from the gutta percha and sealer compromising the quality of those images as regards root filling evaluations.

**Figure 17:**
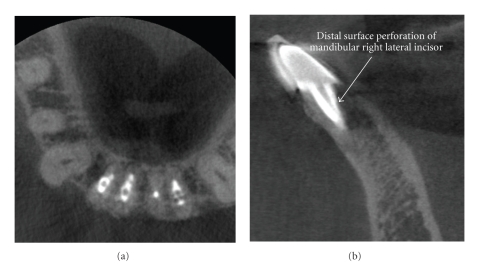
Iatrogenic perforative defects can be difficult to confirm by periapical radiography alone. This patient was referred for evaluation and possible endodontic revision of the mandibular right lateral incisor because of chronic sensitivity to occlusal forces. CBCT images ((a) axial, (b) sagittal) demonstrate a mid-root post perforation.

The utility of CBCT in determining the precise nature of a perforation and the role of this on subsequent treatment has been illustrated by Young (Figure 17) [81]. 

Endodontic surgery is often complicated in the posterior teeth by their proximity to anatomical structures. The mandibular teeth can be close to the mandibular canal while maxillary molars are often close to the maxillary sinus. CBCT imaging provides several advantages for preoperative treatment planning especially in maxillary posterior teeth with apical pathology [82]. Rigolone et al. [83] first described the value of CBCT in planning for endodontic surgery. They imaged 43 maxillary first molars on 31 patients referred for retreatment and measured the mean distance of the palatine root from the external vestibular cortex (Mean; 9.73 mm) and the frequency that the maxillary sinus lateral recess lays between the roots (25%) to evaluate the ability to surgically approach the palatal root of a maxillary molar from a vestibular access as opposed to the more difficult palatal access. They concluded that CBCT may play an important role in optimizing palatine root apicoectomy via directing surgery through vestibular access. The importance of CBCT for apical surgery of teeth adjacent to the maxillary sinus has subsequently been illustrated by Nakata et al. [56] who presented a case report localizing the presence of a periradicular lesion to a specific root and Tsurumachi and Honda [84] who described the use of CBCT in localizing a fractured endodontic instrument protruding into the maxillary sinus prior to periapical surgery. Most recently Low et al. [51] compared the preoperative findings obtained from periapical radiography and CBCT of 2 observers in the diagnosis of posterior maxillary teeth (37 premolars and 37 molars—a total of 156 roots) referred for possible apical surgery. They found that CBCT demonstrated significantly more lesions (34%) than conventional radiography. They also reported that numerous additional clinically relevant findings were seen significantly more frequently in CBCT images including expansion of lesions into the maxillary sinus, sinus membrane thickening, and missed canals. 

## 6. Conclusion

Conventional intraoral radiography provides clinicians with an accessible, cost effective, high-resolution imaging modality that continues to be of value in endodontic therapy. There are, however, specific situations, both pre- and postoperatively, where the understanding of spatial relationships afforded by CBCT facilitates diagnosis and influences treatment. The usefulness of CBCT imaging can no longer be disputed—CBCT is a useful task specific imaging modality and an important technology in comprehensive endodontic evaluation.

## Figures and Tables

**Table 1:** Current commercially available CBCT equipment.

Unit	Model(s)	Manufacturer/Distributor
Accuitomo	3D Accuitomo—XYZ Slice View Tomograph/Veraviewpacs 3D	J. Morita Mfg. Corp., Kyoto, Japan
Asahi Roentgen	PSR 9000N (Alphard 3030)	Asahi Roentgen, Kyoto, Japan/Distributed by Belmont, Somerset, NJ, USA
Galileos	Galileos	Sirona Dental Systems, Charlotte, NC, USA
GENDEX	CB 500	Imaging Sciences International, Hatfield, PA, USA/Distributed by Gendex, Chicago, IL, USA
Hitachi	CB MercuRay/CB Throne	Hitachi Medical Corp., Chiba-ken, Japan
iCAT	Classic/Next Generation	Imaging Sciences International, Hatfield, PA, USA
ILUMA	Ultra Cone Beam CT Scanner	IMTEC Imaging Ardmore, OK, USA/Distributed by GE Healthcare, Piscataway, NJ, USA
KaVo	3D eXam	Imaging Sciences International, Hatfield, PA, USA/Distributed by KaVo Dental Corp., Biberach, Germany
KODAK	9000 3D/9500 3D	KODAK Dental Systems, Carestream Health Rochester NY, USA/Distributed exclusively in the USA by PracticeWorks, Atlanta, GA, USA
Newtom	3G/NewTom VG	QR, Inc. Verona, Italy/Dent-X Visionary Imaging, Elmsford, NY, USA
ORION	RCB-888	Ritter Imaging GmbH, Ulm, Germany
Picasso Series	Trio/Pro/Master	E-Woo Technology Co., Ltd/Vatech, Giheung-gu, Korea
PreXion	3D	PreXion, Inc. San Mateo, CA, USA
Promax	3D	Planmeca OY, Helsinki, FInland
Ritter	Orion RCB-888	Ritter Imaging GmbH, Ulm, Germany
Scanora	Scanora 3D CBCT	SOREDEX, Tuusula, Finland
SkyView	3D Panoramic imager	My-Ray Dental Imaging, Cefla Dental Group, Imola, Italy
Suni	3D	Suni Corp., CA, USA
TeraRecon	Fine Cube	Yoshida Dental Mfg. Co. Ltd., Tokyo, Japan/Distributed by TeraRecon, Inc., San Mateo, CA, USA

**Table 2:** Reported Comparative Radiation Effective Dose (*E*_2007_) from Selected Medium and Full FOV CBCT Systems.

				Dose^a^	
			Absolute	Comparative	

CBCT unit	Ref.	Technique	Effective dose^a^ (*μ*Sv)	Digital panoramic equivalent^b^	No. of days of annual per capita background^c^

CB MercuRay	[16]	100 kVp 12-in/9-in/6-in	479/402/369	34/29/26	58/49/45
	[16]	120 kVp 12-in/9-in/6-in	761/680/603	54/49/40	93/83/73
	[17]	Implant mode	511	36.5	62
	[18]	19 cm (Max/Stand)/15 cm Pan/10 cm I	1073/569/560/407	77/41/40/20	131/69/68/50
Galileos	[18]	Default/Maximum	70/128	5/9.1	8.5/15.6
i-Cat Next Gen	[18]	(portrait-17 cm/landscape-13 cm)	74/87	5.3/6.2	9/10.6
i-Cat Classic	[19]	22 cm/13 cm (40 s/10 s)	82/77/48	5.9/5.5/3.4	10/9.4/5.8
	[20]	6 cm Mn (HR/LR)	189/96	13.5/6.86	23/11.7
	[20]	6 cm Mx (HR/LR)	93/59	6.6/4.2	11/7.2
	[20]	22 cm/full	206/134	14.7/9.6	25/16
	[21]	13 cm	61.1	4.4	7.4
Iluma	[18]	20 s/40 s	98/498	7/35.6	11.9/60.6
Newtom 9000	[21]	23 cm	56.2	4	6.9
Newtom 3G	[22]	12-in (Male/female)	93/95	6.6/6.8	11.3/11.6
	[18]	19 cm	68	4.9	8.3
	[19]	6/9/12-in	57/191/30	4/13.6/2.1	6.9/23.2/3.7

^a^Using 2007 ICRP calculations.

^b^Median of published effective dose for digital dental panoramic radiography  =  14 *μ*Sv.

^c^Annual per capita  =  3.0 mSv (3,000 *μ*Sv) per annum.

**Table 3:** Reported Comparative Radiation Effective Dose (*E*_2007_) for Limited, “Focused” or Small FOV CBCT Systems.

				Dose^a^	
			Absolute	Comparative	

CBCT unit	Ref.	Technique	Effective dose^a^ (*μ*Sv)	Digital panoramic equivalent^b^	No. of days of annual per capita background^c^

Kodak 9000 3D	[30]	Mx Post/Mx Ant/Mn Post/Mn Ant	9.8/5.3/38.3/21.7	.7/.4/2.7/1.6	1.2/.6/4.7/2.6
PreXion 3D	[18]	Standard/High Res	189/388	13.5/27.7	23/47
ProMax 3D	[18]	Small/Large	488/652	35/47	59/79
3D Accuitomo	[23]	Ant (4 × 4 cm/6 × 6 cm)	20/43	1.4/3.1	2.5/5.2
	[24]	Min (Mn PM)—Max (Mn 3rd Mol)	11–77	.8–5.5	2.5–5.2
	[19]	Mx (Ant/PM/Mol)	29/44/29	2/3.2/2	3.5/5.3/3.5
		Mn (Ant/PM/Mol)	13/22/29	.9/1.6/2	1.6/2.7/3.5
	[17]	II/FPD Large/FPD Small	30/102/50	2.1/7.3/3.6	3.6/12.4/6
Veraview	[23]	Ant (4 × 4 cm/8 × 4 cm/pan + 4 × 4 cm)	31/40/30	2.2/2.9/2.1	3.8/4.9/3.6
	[25]	4 × 4 cm	2.9	.2	.06

^a^Using 2007 ICRP calculations.

^b^Median of published effective dose for digital dental panoramic radiography  =  14 *μ*Sv.

^c^Annual per capita  =  3.0 mSv (3,000 *μ*Sv) per annum.
